# Supplementary material for: Over 1000-fold enhancement of upconversion luminescence using water-dispersible metal-insulator-metal nanostructures
Source: Nat Commun. 2018 Nov 16;9:4828. doi: 10.1038/s41467-018-07284-w (PMC6240118; doi:10.1038/s41467-018-07284-w)
Supplement: Supplementary file 1 — Supplementary Information [file 41467_2018_7284_MOESM1_ESM.pdf]

Supplementary Information

Over 1000-fold enhancement of upconversion luminescence using water-dispersible metal-insulator-metal nanostructures

Das *et al.*

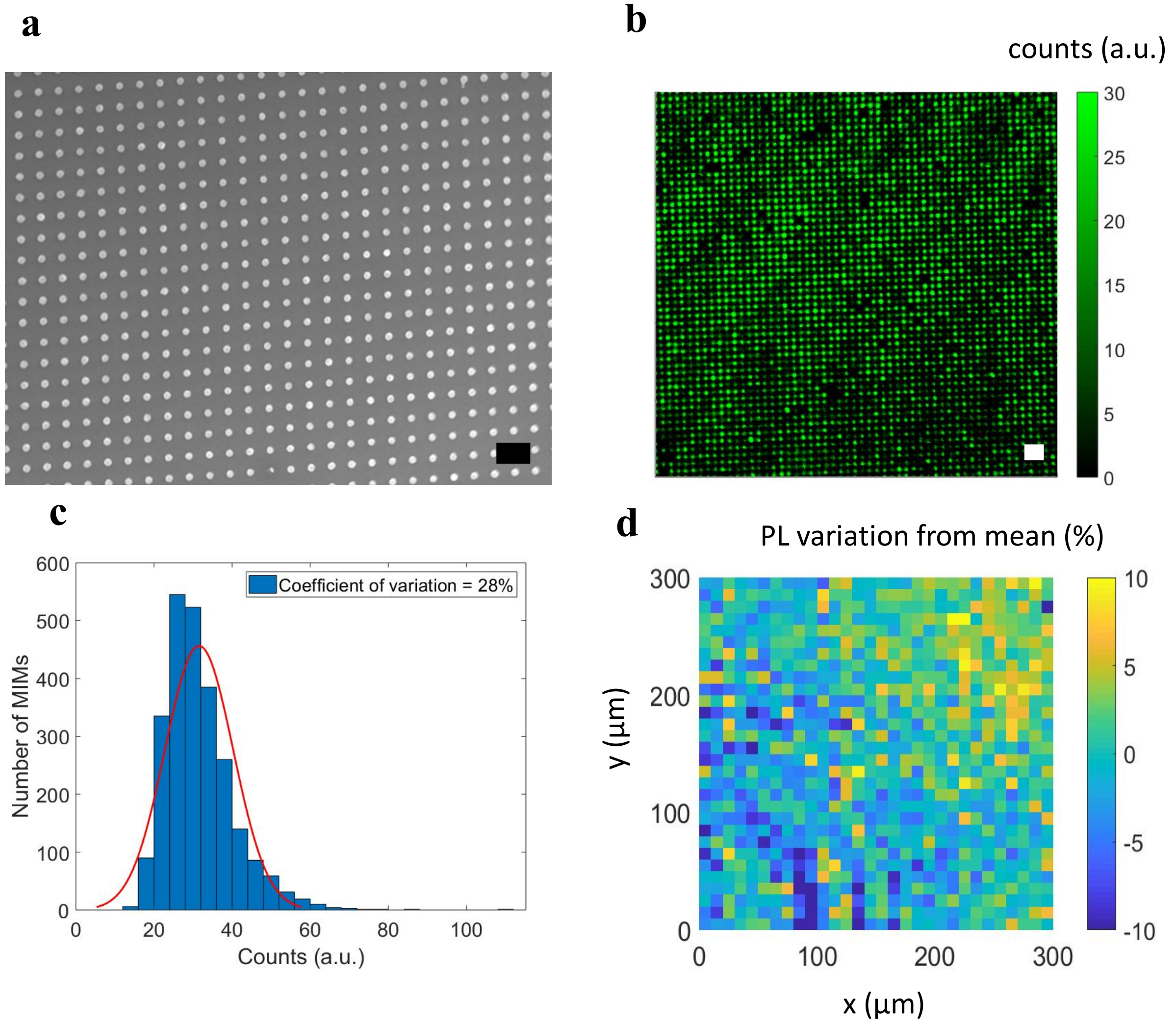

Supplementary Figure 1. Uniformity of metal-insulator-metal (MIM) sample. a Scanning electron microscopy image of MIM sample confirming even deposition and clean resist lift off. Image contains roughly 1000 MIMs. **b** Photoluminescence (PL) from 2500 individual MIMs collected with high numerical aperture (NA) objective lens (NA 1.4). **c.** A Gaussian fit applied to the histogram of the 2500 individual counts shows a coefficient of variation of 28%. **d.** Percent variation from PL intensity averaged over 30 MIMs taken over a 300 μm x 300 μm square of MIMs with 10 μm step size. PL variation is within 10 percent of average value over roughly 100000 MIMs. Scale bar: **a** 1 μm; **b** 2 μm.

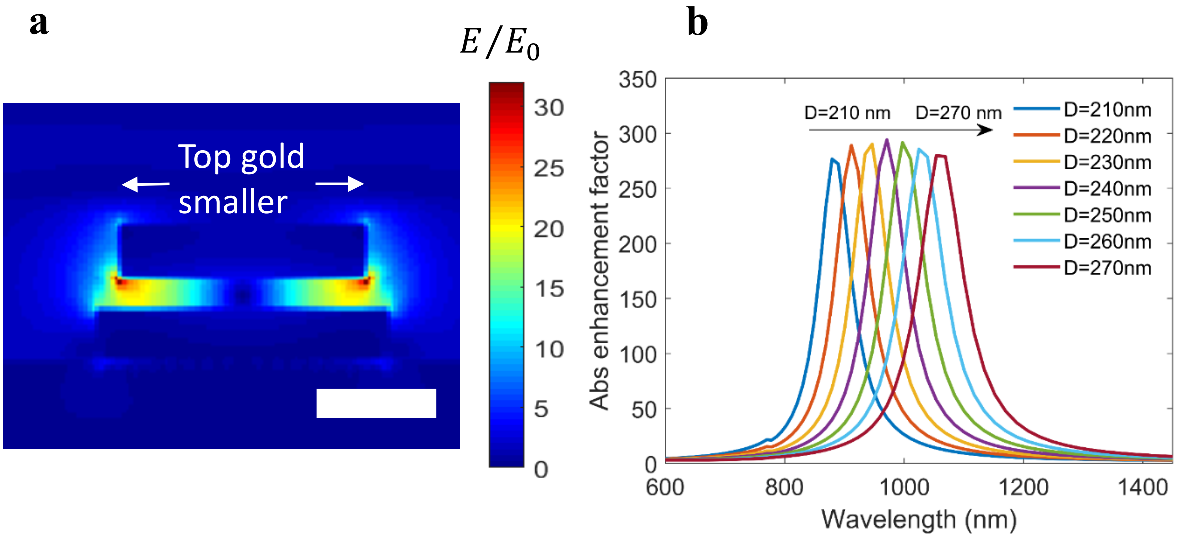

Supplementary Figure 2. Simulated enhancement of fabricated metal-insulator-metal (MIM) structure. a. Simulated field profile of fabricated structure with top metal layer 85% smaller than bottom metal layer for 980 nm normal incidence plane wave excitation. The mode profile is similar to the optimally designed structure with a null in the center and large field intensity throughout the rest of the insulator layer. **b.** Simulated diameter dependence of plasmonic resonance for MIM structure with smaller top gold layer. The structure shows similar diameter dependence to the optimally designed structure, with larger diameters redshifting the resonance. The absorption enhancement at resonance is as large as the original design. Scale bar: **a** 100 nm.

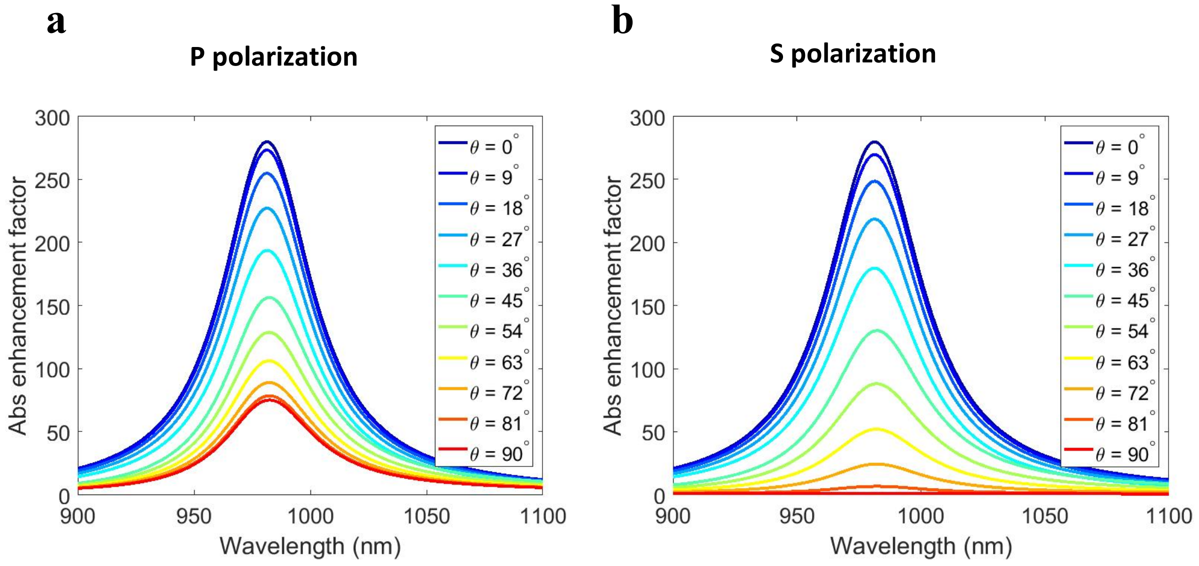

Supplementary Figure 3. Simulated metal-insulator-metal (MIM) enhancement versus incident angle. a. Absorption enhancement as a function of incident angle θ measured from the z axis for p polarization (E field along xz plane). E field for the MIM plasmonic mode is primarily in the z direction so there is still enhancement for excitation along the x axis due to mode overlap (Incident E field at $\theta = 90°$ points along z axis) **b.** Absorption enhancement as a function of incident angle θ measured from the z axis for s polarization (E field tangential to xy plane). Enhancement for excitation along the x axis is 0 for this polarization due to poor mode overlap. Averaging over all angles and polarizations, we calculate an expected enhancement of 112 which is 41% of the enhancement predicted for normal incidence.

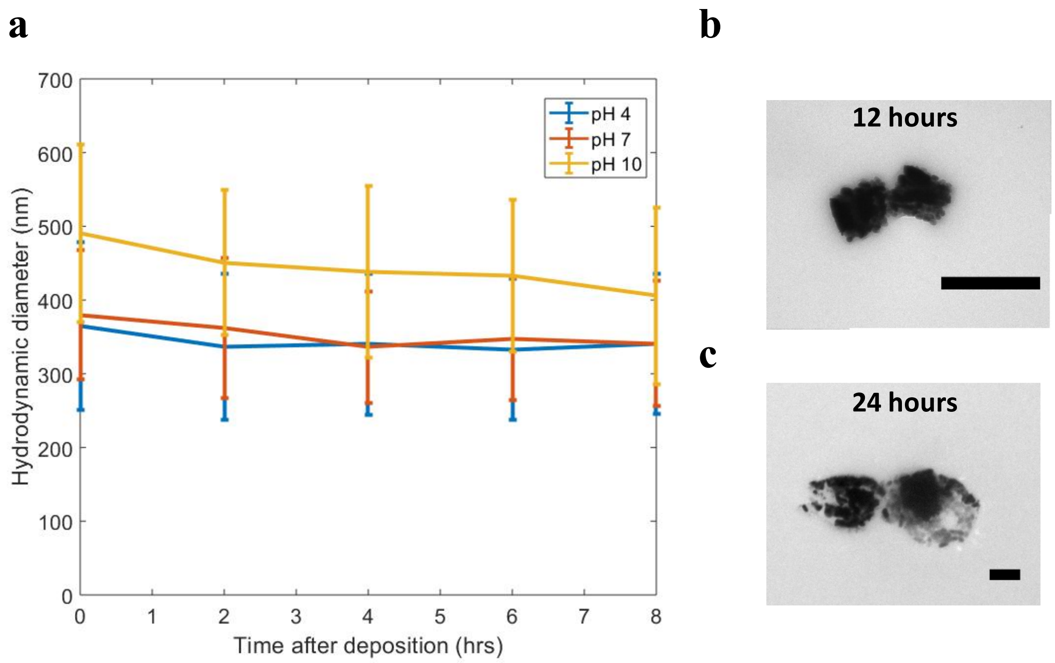

Supplementary Figure 4. Colloidal stability of nanostructures over time. a Physical and chemical stability of dispersed metal-insulator-metal nanostructures was tested by monitoring dynamic light scattering measurements of various buffer solutions over a 24 hour period. The samples were stable over 8 hours but deteriorated by the 24 hour mark. Standard deviations were calculated by fitting the dynamic light scattering data to a normal distribution. They represent the uncertainty in the hydrodynamic radius of the MIM nanostructures. **b** Transmission electron microscopy (TEM) image confirms colloidal stability up to 12 hours **c** TEM image showing degradation at 24 hours. Scale bars: **b** 500 nm; **c** 100 nm

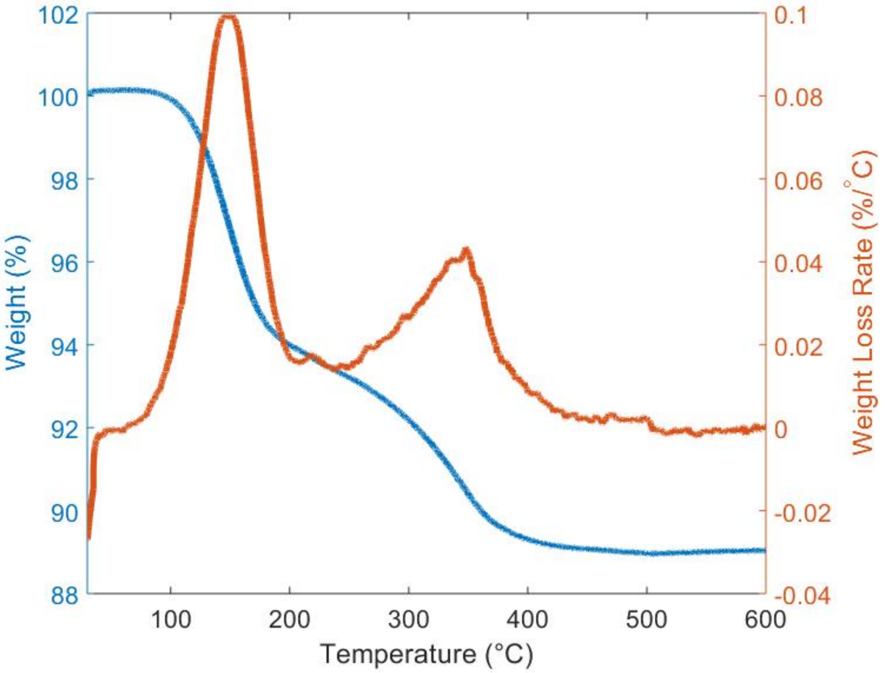

Supplementary Figure 5. Thermal stability of upconversion nanoparticles (UCNPs). Thermogravimetric analysis of UCNPs shows that the metal-insulator-metal nanostructures are stable for temperatures up to 100°C. We observed two major weight loss events occurring at around 150°C and around 350°C. We attribute these events to the evaporation of the residual 1 Octadecene alkene (flash point 155°C) and Oleic acid (boiling point 350°C) that are known to coat the UCNPs in our synthesis process.

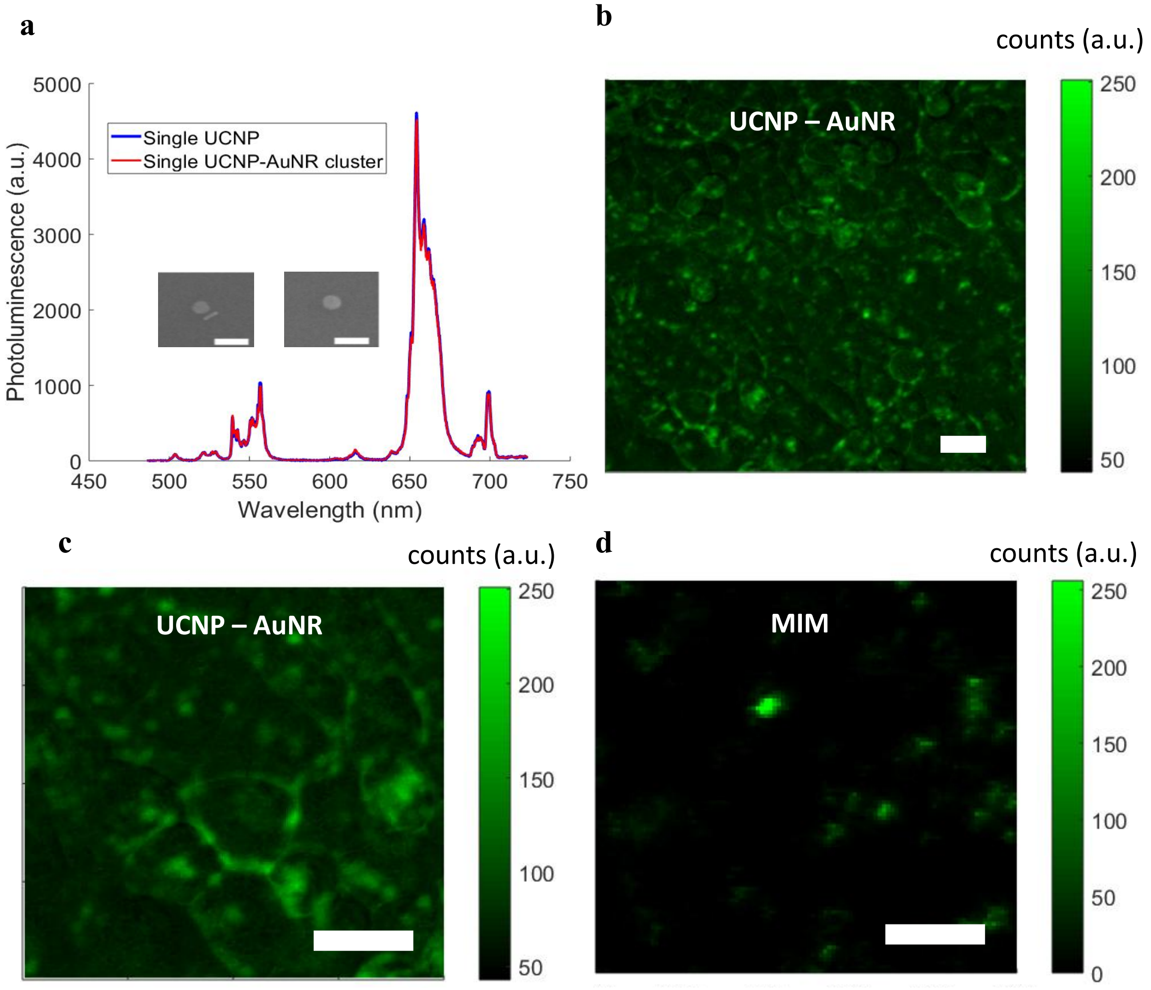

Supplementary Figure 6. Metal-insulator-metal (MIM) nanostructure versus upconversion nanoparticle gold nanorod cluster (UCNP-AuNR) comparison. a. Photoluminescence (PL) from a UCNP conjugated to a gold nanorod that supports a plasmon resonance at 800nm vs bare UCNP. Due to the deliberate detuning of the plasmon resonance away from the emission and absorption wavelengths of UCNP, PL from UCNP-AuNR cluster is similar to PL from bare UCNP and thus can be used as a reference to evaluate in-vitro MIM performance. **b.** Overlay image of brightfield image with the green upconversion photoluminescence image of T24T cells incubated with UCNP-AuNR nanoclusters. **c.** A zoomed in image of the UCNP-AuNR nanocluster upconverted PL image in b and **d.** a zoomed-in image of the MIM upconversion PL image in Figure 5c. After accounting for the acquisition conditions (irradiation power and detector gain), similar levels of brightness were obtained with a 3 orders of magnitude lower concentration of MIMs than UCNP-AuNR clusters. Scale bars: **a** 100 nm; **b-d** 20 µm

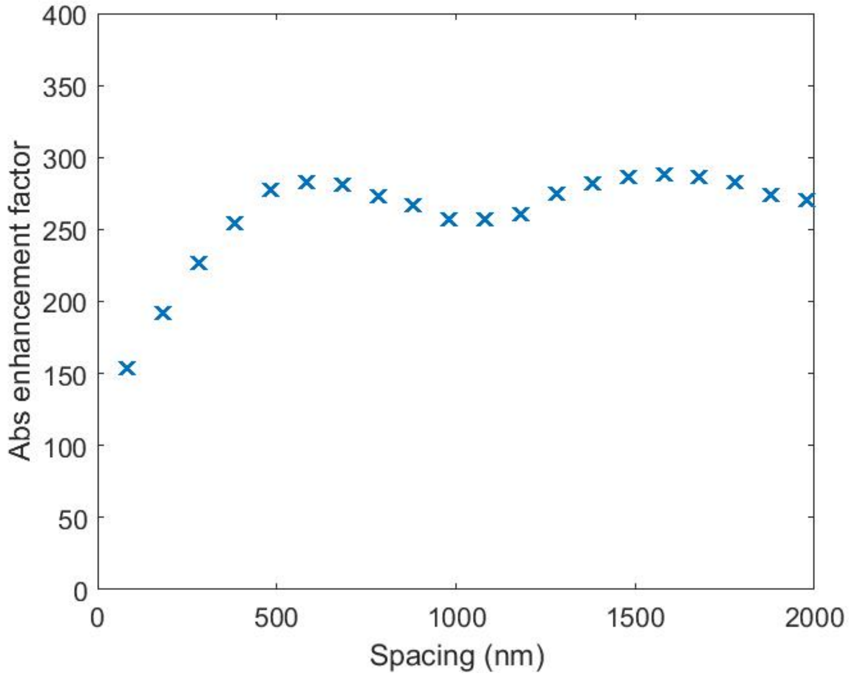

Supplementary Figure 7. Simulated metal-insulator-metal (MIM) enhancement versus separation distance. Simulated absorption enhancement of two MIMs under normal incidence unpolarized excitation as a function of the separation distance between them. For separations larger than 500 nm, the absorption enhancement in the two MIMs is similar to that of a single, isolated MIM, with small variations occurring near integer multiples of the plasmonic wavelength (here 980 nm).

Supplementary Note 1 - Green emission rate formula derivation

For the subsequent rate equation analysis, we ignore the $^4F_{7/2}$ level of Er^{3+} as the relaxation from the $^4F_{7/2}$ level to the $^2H_{11/2}$ and $^4S_{3/2}$ levels is known to be extremely fast. We also assume that the $^2H_{11/2}$ and $^4S_{3/2}$ levels are close enough to be considered a single level. The complete set of rate equations can be written as[1]:

$$\frac{dN_{\mathrm{D1}}}{dt} = \sigma\Phi N_{\mathrm{D0}} - W_{\mathrm{D10}}N_{\mathrm{D1}} + c_{\mathrm{Bd2}}N_{\mathrm{A2}}N_{\mathrm{D0}} - c_{\mathrm{Fd2}}N_{\mathrm{D1}}N_{\mathrm{A0}} - c_{\mathrm{d3}}N_{\mathrm{A1}}N_{\mathrm{D1}} - c_{\mathrm{d4}}N_{\mathrm{D1}}N_{\mathrm{A2}} \quad (1)$$

$$\frac{dN_{\mathrm{A1}}}{dt} = W_{\mathrm{A21}}N_{\mathrm{A2}} - W_{\mathrm{A10}}N_{\mathrm{A1}} - c_{\mathrm{d3}}N_{\mathrm{D1}}N_{\mathrm{A1}} \quad (2)$$

$$\frac{dN_{\mathrm{A2}}}{dt} = c_{\mathrm{Fd2}}N_{\mathrm{D1}}N_{\mathrm{A0}} - c_{\mathrm{Bd2}}N_{\mathrm{A2}}N_{\mathrm{D0}} - c_{\mathrm{d4}}N_{\mathrm{D1}}N_{\mathrm{A2}} - W_{\mathrm{A20}}N_{\mathrm{A2}} - W_{\mathrm{A21}}N_{\mathrm{A2}} \quad (3)$$

$$\frac{dN_{\mathrm{A3}}}{dt} = W_{\mathrm{A43}}N_{\mathrm{A4}} + c_{\mathrm{d3}}N_{\mathrm{A1}}N_{\mathrm{D1}} - W_{\mathrm{A30}}N_{\mathrm{A3}} \quad (4)$$

$$\frac{dN_{\mathrm{A4}}}{dt} = c_{\mathrm{d4}}N_{\mathrm{D1}}N_{\mathrm{A2}} - W_{\mathrm{A4}}N_{\mathrm{A4}} \quad (5)$$

$$N_{\mathrm{D}} = N_{\mathrm{D0}} + N_{\mathrm{D1}} \quad (6)$$

$$N_{\mathrm{A}} = N_{\mathrm{A0}} + N_{\mathrm{A1}} + N_{\mathrm{A2}} + N_{\mathrm{A3}} + N_{\mathrm{A4}} \quad (7)$$

In the steady state, under continuous-wave excitation, all time derivatives are zero. In the weak excitation limit, the densities of excited ions would be small and the decay processes are more efficient than upconversion. Thus, we may ignore $c_{\mathrm{d3}}N_{\mathrm{D1}}N_{\mathrm{A2}}$ and $c_{\mathrm{d4}}N_{\mathrm{D1}}N_{\mathrm{A2}}$ terms in equations (1)-(3). Also the ground state populations of the donor and acceptor should be equal to the doping densities. So equations (1), (2), (6) and (7) are simplified to (8) - (12).

$$\frac{dN_{\mathrm{D1}}}{dt} = \sigma\Phi N_{\mathrm{D0}} - W_{\mathrm{D10}}N_{\mathrm{D1}} + c_{\mathrm{Bd2}}N_{\mathrm{A2}}N_{\mathrm{D0}} - c_{\mathrm{Fd2}}N_{\mathrm{D1}}N_{\mathrm{A0}} \quad (8)$$

$$\frac{dN_{\mathrm{A1}}}{dt} = W_{\mathrm{A21}}N_{\mathrm{A2}} - W_{\mathrm{A10}}N_{\mathrm{A1}} \quad (9)$$

$$\frac{dN_{\mathrm{A2}}}{dt} = c_{\mathrm{Fd2}}N_{\mathrm{D1}}N_{\mathrm{A0}} - c_{\mathrm{Bd2}}N_{\mathrm{A2}}N_{\mathrm{D0}} - W_{\mathrm{A20}}N_{\mathrm{A2}} - W_{\mathrm{A21}}N_{\mathrm{A2}} \quad (10)$$

$$N_{\mathrm{D}} = N_{\mathrm{D0}} \quad (11)$$

$$N_{\mathrm{A}} = N_{\mathrm{A0}} \quad (12)$$

Substitute (10) into (8), we obtain,

$$0 = \sigma\Phi N_{\mathrm{D0}} - W_{\mathrm{D10}}N_{\mathrm{D1}} - W_{\mathrm{A20}}N_{\mathrm{A2}} - W_{\mathrm{A21}}N_{\mathrm{A2}} = \sigma\Phi N_{\mathrm{D}} - W_{\mathrm{D10}}N_{\mathrm{D1}} - W_{\mathrm{A2}}N_{\mathrm{A2}} \quad (13)$$

where $W_{\mathrm{A2}} = W_{\mathrm{A20}} + W_{\mathrm{A21}}$. From equation (10), we get

$$N_{\mathrm{D1}} = \frac{c_{\mathrm{Bd2}}N_{\mathrm{D}} + W_{\mathrm{A2}}}{c_{\mathrm{Fd2}}N_{\mathrm{A}}}N_{\mathrm{A2}} \quad (14)$$

Combine equation (13) and (14), the formula of N_{A2} is shown in equation (15).

$$N_{A2} = \frac{\sigma\Phi N_D}{W_{A2}+W_{D10}(c_{Bd2}N_D+W_{A2})/c_{Fd2}N_A} \tag{15}$$

In addition, from equation (9), population of A1 level can be written as a function of N_{A2} .

$$N_{A1} = \frac{W_{A21}}{W_{A10}}N_{A2} \tag{16}$$

The green photon emission rate is defined by the radiative decay rate (W_{A40}) from A4 level to A0 level times the population density of A4 level. Together with the above derivation result, the green photon emission rate is written as

$$\Phi_G = W_{A40}N_{A4} = \frac{W_{A40}}{W_{A4}}c_{d4}N_{D1}N_{A2} \tag{17}$$

Substituting equations (14) and (15) into (17) gives us

$$\Phi_W = \frac{W_{A40}}{W_{A4}}\frac{c_{d4}(c_{Bd2}N_D + W_{A2})N_D^2}{c_{Fd2}N_A\left[W_{A2} + \frac{W_{D10}(c_{Bd2}N_D + W_{A2})}{c_{Fd2}N_A}\right]^2}(\sigma\Phi)^2 \tag{18}$$

It is noted that if W_{A2} is negligible, equation (18) can be further simplified to

$$\Phi_W = \frac{W_{A40}}{W_{A4}}\frac{c_{d4}c_{Fd2}}{c_{Bd2}}\frac{N_A N_D}{W_{D10}^2}(\sigma\Phi)^2 \tag{19}$$

as reported before[1]. Using a back energy transfer coefficient[3] c_{Bd2} of 1.16×10^{-16} cm^3 s^{-1} and a doping density N_D of 1.98×10^{21} cm^{-3}, the back energy transfer term, $c_{Bd2}N_D$, is calculated to be 2.30×10^5 s^{-1}. As reported in the Results section, W_{A2} was found to be 7.05×10^4 s^{-1} for the MIM structure and 1.07×10^4 s^{-1} for the reference, which are 30% and 5% of the back energy transfer term, respectively. They are therefore not small enough to be ignored in the current case and we thus use the more complex full expression given in equation (18).

For the strong excitation limit, we rearrange equations (1-3) as follows

$$0 = \sigma\Phi N_{D0} - W_{D10}N_{D1} - c_{d3}N_{D1}N_{A1} - 2c_{d4}N_{D1}N_{A2} - W_{A20}N_{A2} - W_{A21}N_{A2} \tag{20}$$

$$0 = \sigma\Phi N_{D0} - W_{D10}N_{D1} - 2W_{A21}N_{A2} - W_{A20}N_{A2} - W_{A10}N_{A1} - 2c_{d4}N_{D1}N_{A2} \tag{21}$$

In the strong excitation limit, the energy transfer processes dominate over the decay processes and we thus ignore all decay terms and only retain energy transfer terms. From equation (21), we find

$$0 = \sigma\Phi N_{D0} - 2c_{d4}N_{D1}N_{A2} \tag{22}$$

Plugging this into equation (17), we derive the expected green photon emission rate un strong excitation which is given by

$$\Phi_{\mathrm{S}} = \frac{W_{\mathrm{A40}}}{W_{\mathrm{A4}}}\frac{N_{\mathrm{D0}}}{2}\sigma\Phi \quad (23)$$

Supplementary Note 2 - Transient near-infrared (NIR) photoluminescence (PL)

It has been shown[2] that the NIR PL decay follows rate equation (24) which can be obtained directly from equation (8) for pulsed excitation conditions.

$$\frac{dN_{\mathrm{D1}}}{dt} = -W_{\mathrm{D10}}N_{\mathrm{D1}} - c_{\mathrm{d3}}N_{\mathrm{A1}}N_{\mathrm{D1}} - 2c_{\mathrm{d4}}N_{\mathrm{D1}}N_{\mathrm{A2}} \quad (24)$$

And the analytical solution of N_{D1} is written as

$$N_{\mathrm{D1}}(t) = \exp\left[-(W_{\mathrm{D10}} + 2c_{\mathrm{d4}}N_{\mathrm{A2}} + c_{\mathrm{d3}}N_{\mathrm{A1}})t\right] \quad (25)$$

where W_{D10} is the intrinsic donor rates, and $2c_{\mathrm{d4}}N_{\mathrm{A2}} + c_{\mathrm{d3}}N_{\mathrm{A1}}$ is the energy transfer upconversion (ETU) rate. Then, the total decay rate W_{D1} of NIR PL is given as

$$W_{\mathrm{D1}} = W_{\mathrm{D10}} + 2c_{\mathrm{d4}}N_{\mathrm{A2}} + c_{\mathrm{d3}}N_{\mathrm{A1}} \quad (26)$$

In the weak excitation regime, the excited state population is small and consequently ETU rate is small. Thus, the decay rate is dominated by W_{D10}. In the strong excitation regime, both intrinsic decay rate, W_{D10}, and ETU rate, $2c_{\mathrm{d4}}N_{\mathrm{A2}} + c_{\mathrm{d3}}N_{\mathrm{A1}}$, contribute to the total decay rate. By fitting the transient NIR PL with single exponential function, we can get W_{D10} and W_{D1} under weak and strong excitation condition, respectively.

Supplementary Note 3 - Transient green photoluminescence (PL)

The metal quenching effect directly has an impact on green emission, which can be demonstrated by analyzing the green decay rates of MIM and reference sample. The transient green PL was monitored at 550nm. It has been shown[2] that the green PL should follow the normalized decay expression

$$N_{\mathrm{A4}}(t) = \frac{W_{\mathrm{A4}}}{W_{\mathrm{A4}} - W_{\mathrm{total}}}\left[e^{-W_{\mathrm{total}}t} - \frac{W_{\mathrm{total}}}{W_{\mathrm{A4}}}e^{-W_{\mathrm{A4}}t}\right] \quad (27)$$

where W_{total} is the sum of decay rates W_{D1} and W_{A2}. Since it is not possible to determine W_{A2} independently, W_{A4} and W_{total} are both obtained by fitting the decay of green luminescence.

Supplementary References

1. Lu, D. *et al.* Plasmon Enhancement Mechanism for the Upconversion Processes in $NaYF_4$:Yb^{3+},Er^{3+} Nanoparticles: Maxwell versus Förster. *ACS Nano* **8,** 7780–7792 (2014).
2. Lu, D., Mao, C., Cho, S. K., Ahn, S. & Park, W. Experimental demonstration of plasmon enhanced energy transfer rate in $NaYF_4$:Yb^{3+},Er^{3+} upconversion nanoparticles. *Sci. Rep.* **6,** 18894 (2016).
3. Hwang, B.-C. *et al.* Cooperative upconversion and energy transfer of new high Er^{3+}- and Yb^{3+}–Er^{3+}-doped phosphate glasses. *J. Opt. Soc. Am. B* **17,** 833–839 (2000).
